# gcPathogen: a comprehensive genomic resource of human pathogens for public health

**DOI:** 10.1093/nar/gkad875

**Published:** 2023-10-18

**Authors:** Chongye Guo, Qi Chen, Guomei Fan, Yan Sun, Jingyi Nie, Zhihong Shen, Zhen Meng, Yuanchun Zhou, Shiwen Li, Shuai Wang, Juncai Ma, Qinglan Sun, Linhuan Wu

**Affiliations:** Microbial Resource and Big Data Center, Institute of Microbiology, Chinese Academy of Sciences, Beijing 100101, China; Chinese National Microbiology Data Center (NMDC), Beijing 100101, China; Microbial Resource and Big Data Center, Institute of Microbiology, Chinese Academy of Sciences, Beijing 100101, China; Chinese National Microbiology Data Center (NMDC), Beijing 100101, China; Microbial Resource and Big Data Center, Institute of Microbiology, Chinese Academy of Sciences, Beijing 100101, China; Chinese National Microbiology Data Center (NMDC), Beijing 100101, China; Microbial Resource and Big Data Center, Institute of Microbiology, Chinese Academy of Sciences, Beijing 100101, China; Chinese National Microbiology Data Center (NMDC), Beijing 100101, China; Microbial Resource and Big Data Center, Institute of Microbiology, Chinese Academy of Sciences, Beijing 100101, China; Chinese National Microbiology Data Center (NMDC), Beijing 100101, China; Computer Network Information Center, Chinese Academy of Sciences, Beijing 100190, China; Computer Network Information Center, Chinese Academy of Sciences, Beijing 100190, China; Computer Network Information Center, Chinese Academy of Sciences, Beijing 100190, China; Microbial Resource and Big Data Center, Institute of Microbiology, Chinese Academy of Sciences, Beijing 100101, China; Chinese National Microbiology Data Center (NMDC), Beijing 100101, China; Microbial Resource and Big Data Center, Institute of Microbiology, Chinese Academy of Sciences, Beijing 100101, China; Chinese National Microbiology Data Center (NMDC), Beijing 100101, China; Microbial Resource and Big Data Center, Institute of Microbiology, Chinese Academy of Sciences, Beijing 100101, China; Chinese National Microbiology Data Center (NMDC), Beijing 100101, China; State Key Laboratory of Microbial Resources, Institute of Microbiology, Chinese Academy of Sciences, Beijing 100101, China; Microbial Resource and Big Data Center, Institute of Microbiology, Chinese Academy of Sciences, Beijing 100101, China; Chinese National Microbiology Data Center (NMDC), Beijing 100101, China; Microbial Resource and Big Data Center, Institute of Microbiology, Chinese Academy of Sciences, Beijing 100101, China; Chinese National Microbiology Data Center (NMDC), Beijing 100101, China; State Key Laboratory of Microbial Resources, Institute of Microbiology, Chinese Academy of Sciences, Beijing 100101, China

## Abstract

Here, we present the manually curated Global Catalogue of Pathogens (gcPathogen), an extensive genomic resource designed to facilitate rapid and accurate pathogen analysis, epidemiological exploration and monitoring of antibiotic resistance features and virulence factors. The catalogue seamlessly integrates and analyzes genomic data and associated metadata for human pathogens isolated from infected patients, animal hosts, food and the environment. The pathogen list is supported by evidence from medical or government pathogenic lists and publications. The current version of gcPathogen boasts an impressive collection of 1 164 974 assemblies comprising 986 044 strains from 497 bacterial taxa, 4794 assemblies encompassing 4319 strains from 265 fungal taxa, 89 965 assemblies featuring 13 687 strains from 222 viral taxa, and 646 assemblies including 387 strains from 159 parasitic taxa. Through this database, researchers gain access to a comprehensive ‘one-stop shop’ that facilitates global, long-term public health surveillance while enabling in-depth analysis of genomes, sequence types, antibiotic resistance genes, virulence factors and mobile genetic elements across different countries, diseases and hosts. To access and explore the data and statistics, an interactive web interface has been developed, which can be accessed at https://nmdc.cn/gcpathogen/. This user-friendly platform allows seamless querying and exploration of the extensive information housed within the gcPathogen database.

## Graphical Abstract

**Graphical Abstract:**
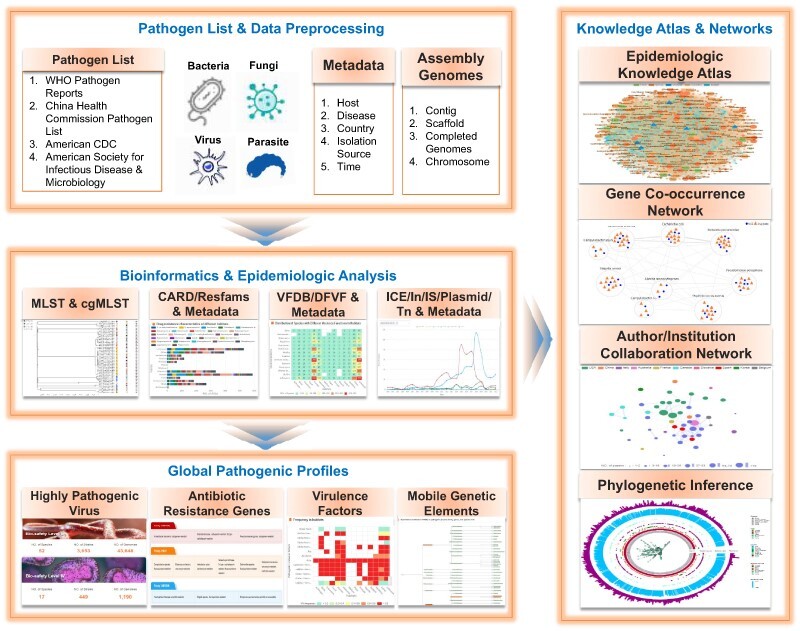

## Introduction

Infectious diseases are one of the major threats to global public health. The rapid accumulation of genomic sequence data has become increasingly crucial in supporting infectious disease surveillance and epidemiological investigations. Whole-genome sequencing (WGS) data based on typing methods, such as core genome multilocus sequence typing (cgMLST) (1) and whole genome multilocus sequence typing (wgMLST) (2), are widely employed due to their advantages in speed, cost-effectiveness and high resolution, providing ample information for surveillance, outbreak investigations, source attribution and evolutionary studies (2,3).

The challenge of antibiotic resistance in pathogens has profound implications for human health. Identifying ARGs (antibiotic resistance genes) and tracking their spread among different pathogens or subtypes of the same pathogen can offer valuable insights for drug development, disease prevention and treatment (4,5). Meanwhile, virulence factors (VF) enable pathogens to infect their hosts and cause disease. Identifying and analyzing these factors can aid in determining the occurrence of large-scale disease outbreaks. Both ARGs and virulence factors are often acquired through horizontal transfer with MGEs (mobile genetic elements) (6).

Although the availability of WGS data supports accurate pathogen identification and deeper insights into VF, ARG and MGE diversity, current genomic resources do not encompass the full spectrum of human pathogens, restricting studies to only a subset of species. To address this limitation, a comprehensive genomic resource encompassing all known human pathogens, complemented by carefully curated metadata, would greatly enhance global investigations across long timescales and significantly improve the control and treatment of infectious diseases. Additionally, a substantial portion of genomic data interpretation and decision-making relies on data visualization. However, current platforms often lack the integration of genomic and epidemiologic data to create interactive visualizations (7).

To bridge these gaps, we have developed gcPathogen (Global Catalogue of Pathogens), a comprehensive genomic resource that collates, integrates and analyzes data from human pathogens isolated from infected patients, animal hosts and environmental samples worldwide. The database relies on manually curated, high-quality data, enabling genomic analysis, MLST, cgMLST and profiling of ARGs, VFs and MGEs, all within interactive interfaces. This powerful tool empowers experienced researchers to conduct more extensive pathogen studies, while also democratizing sophisticated analyses, making them accessible to public health specialists, clinicians and other non-experts in genomics or bioinformatics.

## Database interface and features

### Webpage of the pathogens

**Figure 1.**
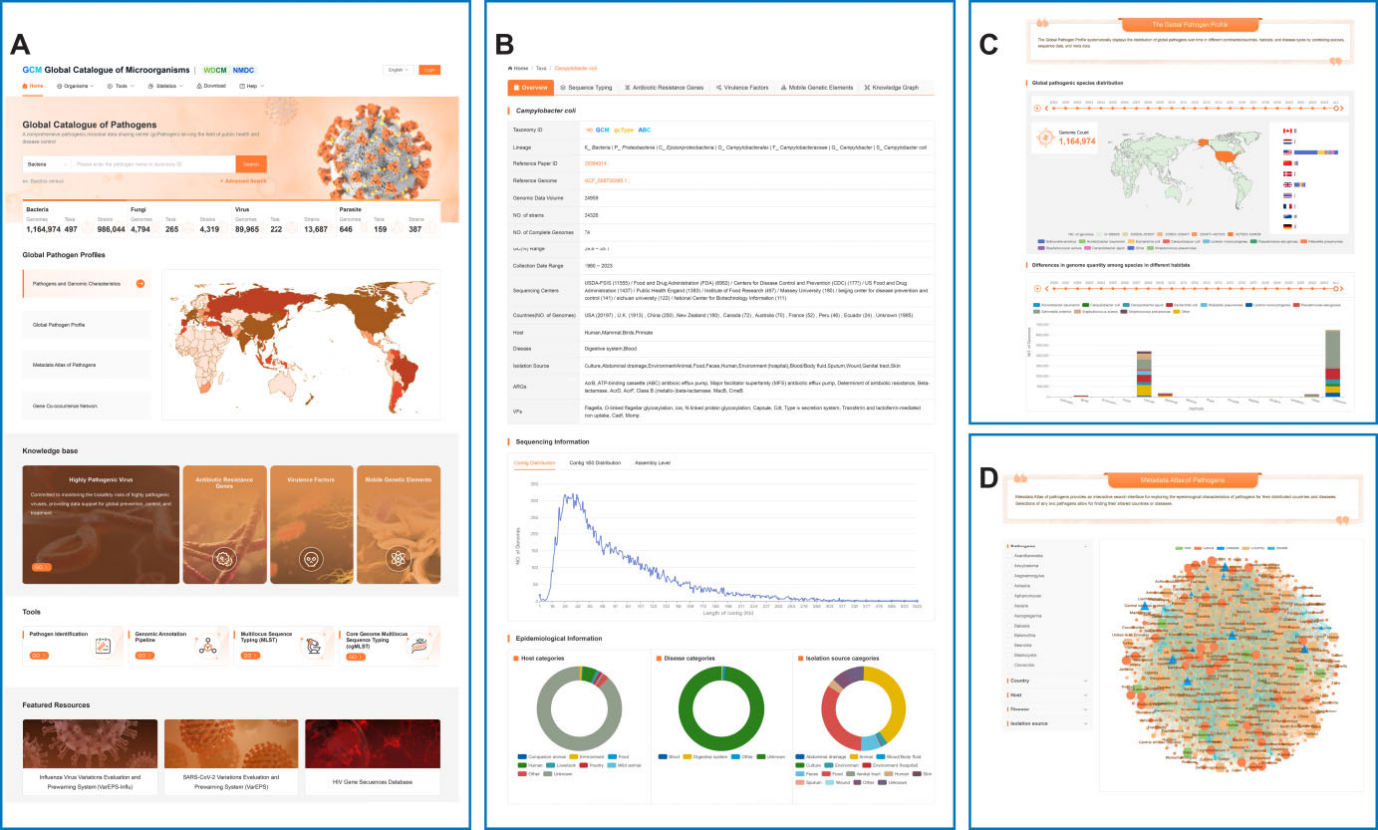
Features of the gcPathogen website. (**A**) Homepage of gcPathogen. (**B**) An overview of pathogen species. (**C**) Distribution maps of global pathogens, Antibiotic Resistance Genes and Virulence factors. (**D**) Schematic illustration of the knowledge graph of pathogens and their curated metadata.

In gcPathogen, we have organized DNA sequence assemblies, along with the corresponding manually curated metadata and analysis results for bacteria, fungi, viruses and parasites by taxon (8), encompassing genera, species, subspecies, or serotypes (Figure 1A). Genomic DNA sequence data of pathogenic microorganisms were collected from public databases and publications.

Each pathogen's webpage is divided into several main sections, including an overview, sequence types (STs and cgMLST), antibiotic resistance genes, virulence factors, mobile genetic elements and a knowledge graph (Figure 1B). The overview section presents essential information on sequence assemblies and corresponding metadata, such as sequencing method, submitters, genome size and reference genome. It also provides a global distribution map, host categories, associated diseases, isolation sources and manually curated text from peer-reviewed publications describing outbreaks or infection cases.

For each pathogen, the STs section provides a summary of available information on all STs in a species, including the number of genomes, strains, ARGs, VFs and MGEs associated with ARGs and VFs. Display options for predominant STs by year or country, as well as a correlation diagram of representative STs, are available. Furthermore, users can select sequence assemblies and conduct cgMLST online, provided the corresponding seed file is accessible.

The ARGs, VFs and MGEs sections offer comprehensive information on all annotated ARGs, VFs and MGEs for each species. The resistance to different types of antibiotics can be visually plotted, along with the distribution of resistance, virulence and mobile genetic elements by country, disease, host and isolation sources.

### Feature resources

Feature resources encompass four sections: Highly Pathogenic Virus, as well as a list of annotated ARGs, VF and MGEs. Highly Pathogenic Virus aims to provide data support for evaluating the risk of outbreaks, cross-species virus transmission and global spread. This section includes viruses classified as biosafety levels III and IV. Statistical analysis on the route of transmission, host, isolation sources and countries is available, alongside a knowledge graph showcasing the cooperation network among authors and institutions extracted from publications.

In the Antibiotic Resistance Genes section, a comprehensive profile of pathogenic ARGs is presented, including all ARGs annotated from pathogenic bacteria, high-frequency ARGs in each species and the main ARGs from the WHO global priority list of antibiotic-resistant bacteria (9). Tables and graphs are employed to analyze these ARGs with respect to their countries, species, habitats and associated MGEs. Similarly, VFs and their epidemiological data are integrated and analyzed, using interactive visualization. The MGE list part encompasses five kinds of MGEs, including insertion sequences (IS), integrative conjugative elements (ICE), integrons (IN), plasmids and transposons. These MGEs are analyzed based on their taxonomic distribution, carried ARGs and VFs, and isolation habitats, aiming to understand the occurrence and spread of acquired resistance and virulence of pathogens mediated by MGEs.

### Statistic summary and database search

The ‘pathogens and genomic characteristics’ page provides a statistical summary of the numbers of bacteria, fungi, viruses and parasites, along with their species distribution, data volume and the submitters of the largest amounts of data for each pathogen type. Global distributions of all pathogens, ARGs, VFs and MGEs can be displayed by year and countries (Figure 1C).

The ‘advanced search’ function allows users to conduct one or multiple criteria searches of manually curated metadata related to (i) pathogens, including species name, date of sampling, country, isolation source, host, disease and biosafety level; (ii) sequence assembly, including sequencing platform, STs, assembly level and number of contigs.

gcPathogen effectively employs semantic web technology to display connections among pathogens, isolation sources, diseases and hosts based on curated metadata (Figure 1D). This ‘knowledge graph’ facilitates connections of disparate types of information for users, enabling a comprehensive view and identification of linkages. Additionally, gcPathogen provides a search interface to explore the connections between any selected pathogens.

### Online data analysis pipelines

gcPathogen seamlessly integrates multiple online tools for rapid genomic analyses. The ‘pathogen identification’ tools combine the 16S rRNA gene sequences, genome Average Nucleotide Identity (ANI) to give species identification results. The ‘genomic annotation pipeline’ efficiently annotates bacterial genomes in gcType (10). The ‘MLST’ pipeline determines STs based on user-submitted genome assemblies using the MLST 2.22.1 against PubMLST typing schemes (11). The ‘cgMLST’ pipeline utilizes chewBBACA 2.0.9 (12) to compare the query assemblies with pre-calculated schema, generating a phylogenetic tree with associated metadata. These integrated pipelines, anchored to high-quality reference datasets, empower users to compare their genomic data with the global repository, effectively detecting outbreaks and tracing their origins.

## Database construction and analytical methods

### Pathogen inventory and data sources

We compiled an extensive inventory of human pathogens based on guidance from reputable sources, including the World Health Organization (http://ghdx.healthdata.org/gbd-2016) (13), the Emerging Infectious Diseases and Pathogens list of the US National Institute of Allergy and Infectious Diseases (https://www.niaid.nih.gov/research/emerging-infectious-diseases-pathogens), the National Health Commission of the People's Republic of China, the US Centers for Disease Control (https://search.cdc.gov/search/index.html?all=pathogen=1#results), the Infectious Diseases Society of America and the American Society for Microbiology (14,15). This inventory was further supplemented with reports of outbreaks and relevant research articles. Additionally, the global priority list of antibiotic-resistant bacteria from the World Health Organization (WHO) (16) guided our research, discovery, and development efforts for new antibiotics.

**Figure 2.**
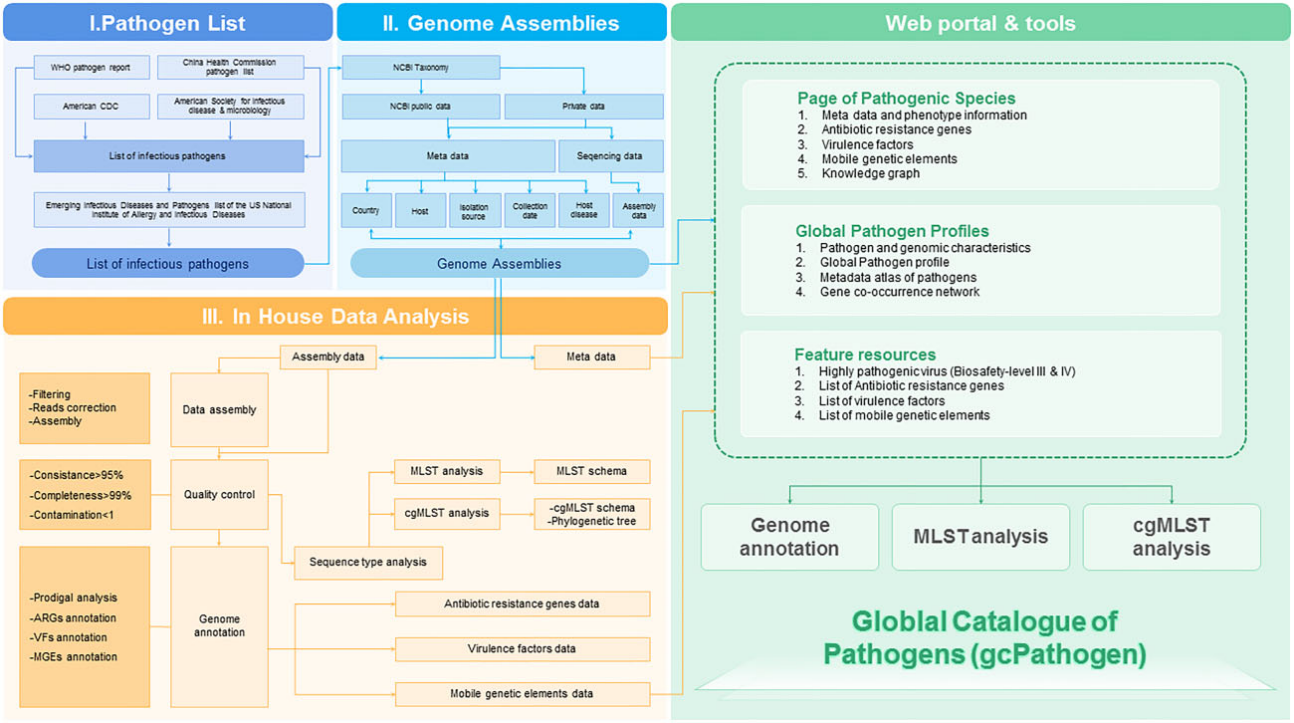
Data processing pipeline used in gcPathogen.

To construct our database, all sequence assemblies for the pathogens in the inventory were retrieved from the genomic database of the National Center for Biotechnology Information (NCBI) (17). The associated metadata from BioSamples (18) were meticulously extracted and integrated. Moreover, the data were referenced to peer-reviewed publications indexed in PubMed (19) (Figure 2).

The current version of this database comprises an impressive collection of 1 164 974 assemblies, encompassing 986 044 strains from 497 bacterial taxa (8), 4794 assemblies featuring 4319 strains from 265 fungal taxa (8), 89 965 assemblies encompassing 13 687 strains from 222 viral taxa (8) and 646 assemblies comprising 387 strains from 159 parasitic taxa (8). This extensive dataset spans 198 countries and regions worldwide, providing a comprehensive epidemiological overview of global pathogens.

### Data quality control

To ensure data integrity, all information uploaded into gcPathogen underwent rigorous quality control checks for consistency, completeness and contamination. Subsequently, the data were analyzed for genes, MLST, cgMLST, ARGs, VFs and MGEs. Only sequence assemblies demonstrating >95% completeness and <5% contamination, as verified by checkM 1.1.3 (20), were retained. Additionally, sequence assemblies were assigned to their respective genera and species based on the NCBI taxonomy. For species determination, assemblies were considered to belong to the same species if their ANI exceeded 95% in the FastANI 1.3 (21) with reference sequences from NCBI’s RefSeq (22). Furthermore, metadata pertaining to host, isolation sources and disease for these assemblies were manually categorized.

### Sequence-based pathogen typing

gcPathogen performed MLST and cgMLST analyses of pathogenic bacteria. Following quality control, assemblies were subjected to MLST analysis (11) to determine their STs. Graphical displays of distribution characteristics of STs based on countries, isolation sources, host and collection date were generated. Additionally, we examined correlations between ARGs/VFs and STs of each species to identify STs-specific ARGs/VFs and their corresponding *p*-values.

For enhanced discrimination and surveillance of pathogen sources, we employed Whole Genome Sequencing (WGS)-based cgMLST. However, as limited cgMLST schema were publicly available for some species, we addressed this challenge by providing pre-calculated cgMLST schemas in gcPathogen. We used the BLAST score ratio-based allele calling algorithm (chewBBACA 2.0.9) (12) to perform cgMLST analysis for all bacterial species with more than 200 assemblies after quality control. In the current version, we included cgMLST schema files for 9 genera and 95 species (encompassing a total of 112 species). These schemas encompass 6 from Enterobase (23), 9 from PubMLST (11) and 9 from PathogenWatch (https://pathogen.watch) (cgMLST schema listed in Supplementary Table S1). Furthermore, in addition to online analysis tools, downloadable cgMLST schema files are also provided, with regular updates as more assemblies become available.

### Annotation of antibiotic resistance genes, virulence factors and mobile genetic elements

Genes in quality-controlled sequence assemblies were predicted using Prokaryotic Dynamic Programming Gene finding Algorithm 2.6.3 (24). Subsequently, resistance genes were annotated based on the Comprehensive Antibiotic Resistance Database (25) using Diamond 0.9.22.123 (26) with coverage exceeding 80%. Additionally, we utilized the Resfams database and HMMER 3.1 (27) to effectively identify even distant homologues of known ARGs (coverage > 80%) (28). For the annotation of virulence factors, we employed the Virulence Factor Database (29) using Diamond with query and subject coverages of 80%. MGEs were annotated based on the methodologies described in the original reference papers (Supplementary Table S2). Furthermore, the MGE-associated ARGs and VFs were determined in accordance with the research principles of Partridge *et al.* (30).

## Data content and case studies

### Taxonomy and spatial distribution of pathogens

**Figure 3.**
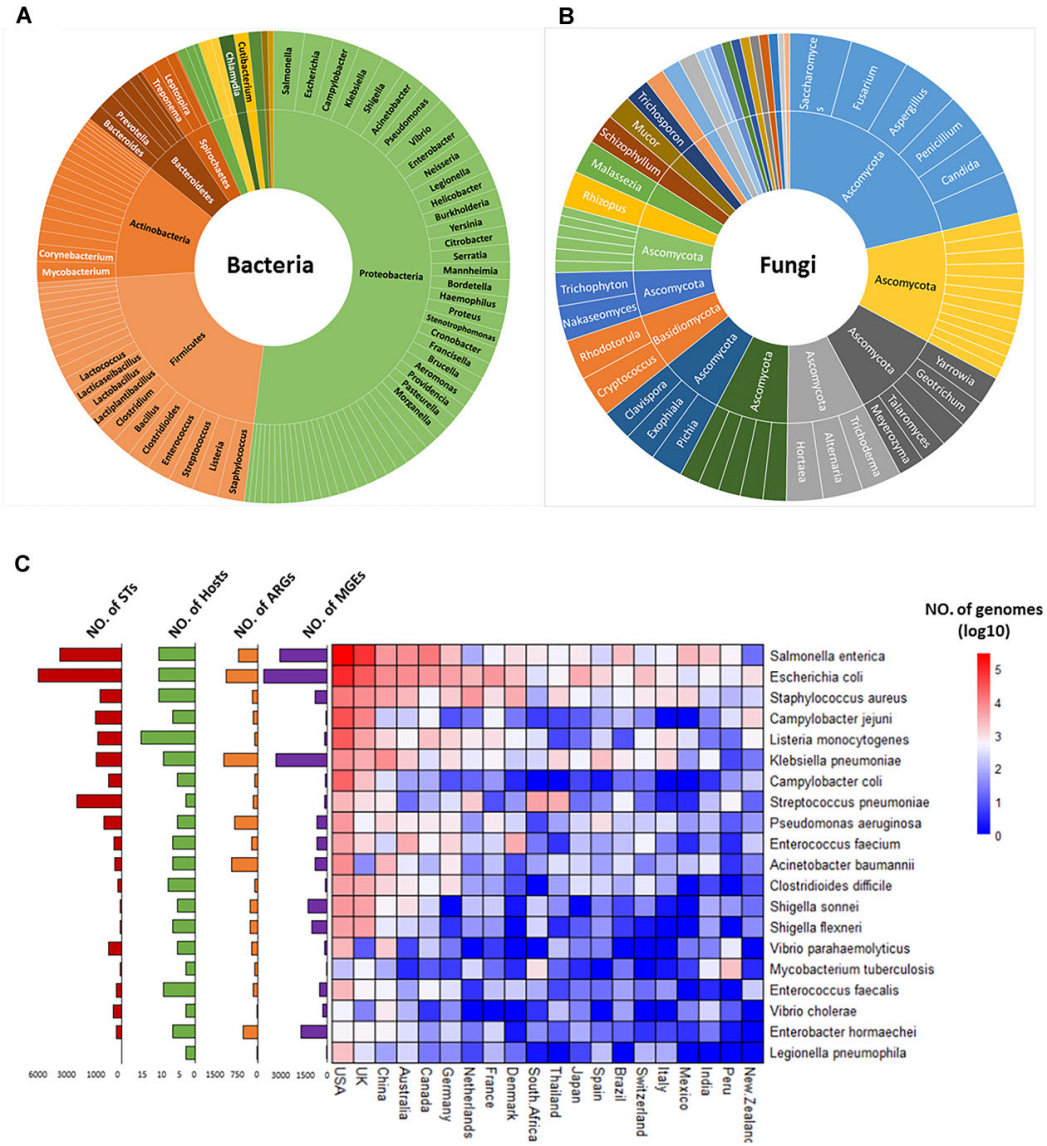
Distribution of bacterial species in gcPathogen. The most abundant genera are (**A**) *Salmonella* in bacteria and (**B**) *Saccharomyces* in fungi. (**C**) The number of STs, host, antibiotic resistance genes (ARGs) and mobile genetic elements (MGEs) among the top 20 bacterial pathogens with the highest data volume. Their distributions in the top 20 countries are also included.

In gcPathogen, the bacteria encompass 13 phyla (Figure 3A): *Proteobacteria* (51 genera), *Firmicutes* (23 genera), *Actinobacteria* (18 genera), *Bacteroidetes* (6 genera), *Spirochaetes* (4 genera), *Bacillota* (3 genera), *Fusobacteria* (2 genera) and six other phyla with one genus each, including *Actinomycetota*, *Bacteroidota*, *Chlamydiae*, *Pseudomonadota*, *Spirochete* and *Tenericutes*. Meanwhile, the fungi comprise five phyla (Figure 3B): *Ascomycota* (61 genera), *Mucoromycota* (11 genera), *Basidiomycota* (9 genera), *Chlorophyta* (1 genus) and *Zoopagomycota* (1 genus). The publication of sequence assemblies from pathogens has seen a sharp increase since 2008, primarily due to the growing application of sequencing technology in pathogen detection. Notably, *Salmonella enterica*, *Escherichia coli* and *Staphylococcus aureus* have exhibited the highest number of sequence submissions from numerous countries, signifying their widespread prevalence (Figure 3C). Conversely, *Vibrio cholerae*, *Vibrio parahaemolyticus* and *Legionella pneumophila* have been associated with local outbreaks. Moreover, *Salmonella enterica*, *Escherichia coli* and *Klebsiella pneumonia* display the highest number of annotated ARGs and MGEs from their genome data. It has been observed that MGEs (including IS, ICE, integrons, transposons and plasmids) contribute to the uptake of exogenous AMR genes in pathogenic bacteria, particularly in *Klebsiella pneumonia* (30). On the other hand, *Pseudomonas aeruginosa* and *Acinetobacter baumannii* exhibit a high number of ARGs, but relatively fewer MGEs. The primary drug resistance mechanism in *Acinetobacter baumannii* is through several resistance islands of varying gene contents, but their association with MGEs remains unreported (31).

**Figure 4.**
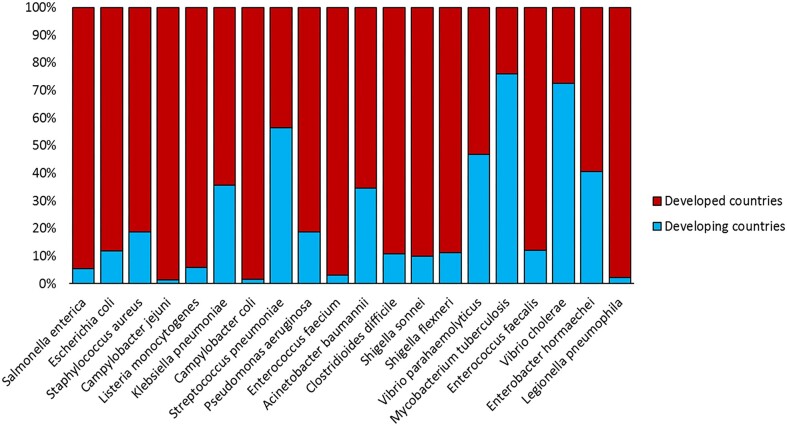
Distribution of the 20 most prevalent bacterial pathogens in developed and developing countries.

The prevalence of the most abundant pathogens in developed and developing countries exhibits significant differences, possibly attributed to varying environments and antibiotic use. For example, *Legionella pneumophila*, *Campylobacter jejuni* and *Campylobacter coli* show much higher prevalence in developed countries than in developing countries (Figure 4). This discrepancy may be related to the development of urban warm water systems for industry and living in developed countries, providing a more suitable environment for *Legionella* (32). Moreover, *Legionella* can contaminate water supply systems in hospitals, posing a major challenge for the outbreak of Legionnaires' disease in developed countries (33). Conversely, its prevalence in developing countries is relatively low. *Mycobacterium tuberculosis* is more prevalent in developing countries, mainly due to its impact on large malnourished and immunocompromised populations, such as HIV patients. In the absence of new anti-tuberculosis drugs, *Mycobacterium tuberculosis* is estimated to infect 40–80% of immunocompromised individuals in developing countries (34).

### Case study: *vibrio parahaemolyticus* typing and antibiotic resistance

**Figure 5.**
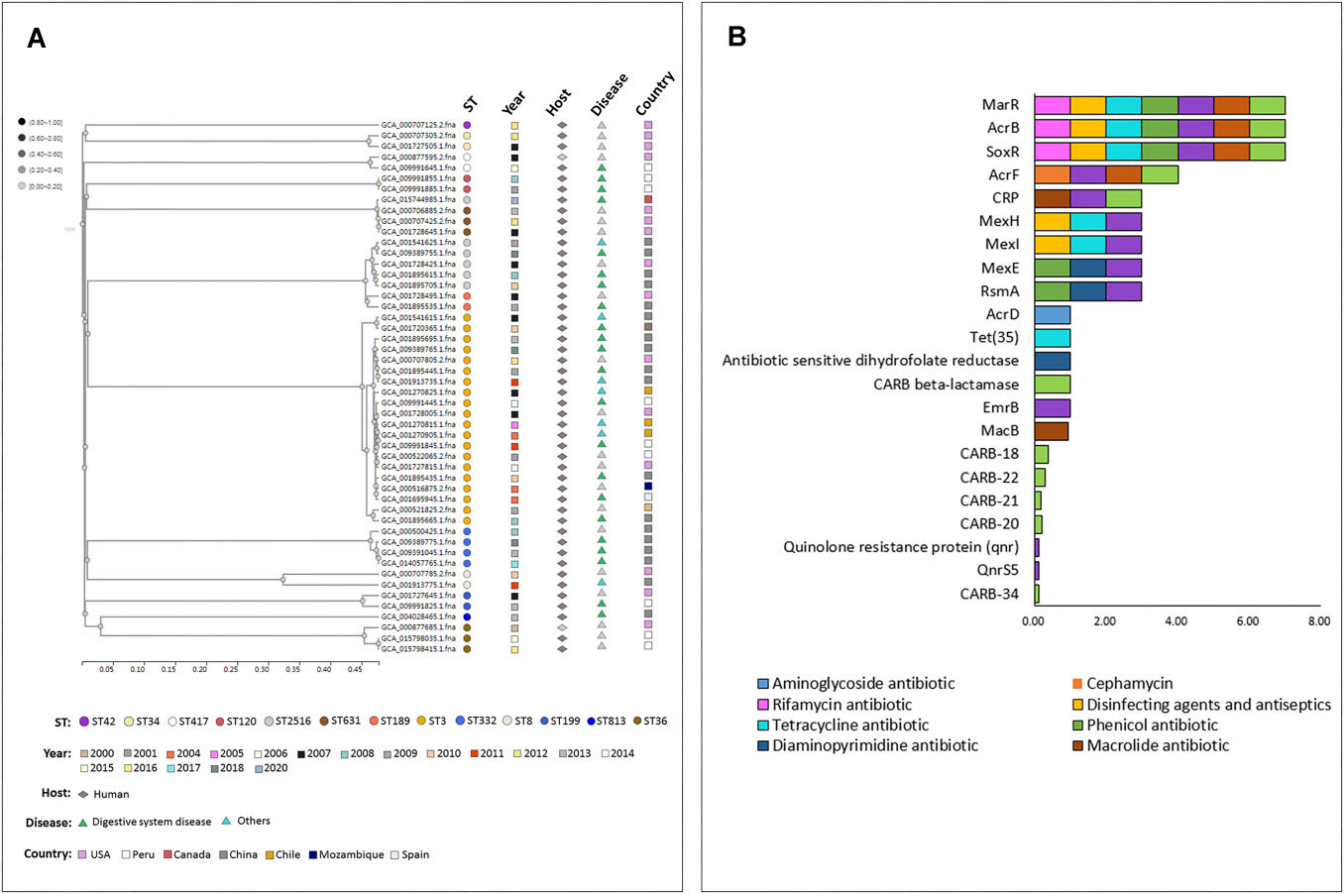
*Vibrio parahaemolyticus* case study based on gcPathogen data analysis. (**A**) Genome data from the top 20 STs, together with their STs, time, host, disease and country information. A phylogenetic tree is drawn and depicted. (**B**) Statistics on the frequency of resistance genes and the characteristics of single and multi-drug resistance to these STs.

Rapid and high-resolution typing can greatly aid epidemiological investigations (35) and the identification of outbreak origins (36). Within gcPathogen, we have identified 1038 STs of Vibrio parahaemolyticus. Among these, ST3 and ST36 are the most prevalent (Figure 5A), with their appearances dating back to before 2000, earlier than other STs in the top 20. ST3 has been found in nearly all countries and is thus classified as a pandemic clone (37). Notably, the profiles of USA, Canada and Peru are similar, but differ from those of China and Thailand (38). Figure 5B demonstrates that these STs contain 22 ARGs resistant to nine drug classes, including nine multi-drug ARGs and 13 single drug ARGs. Particularly, the genes *MarR*, *AcrB* and *SoxB* confer resistance to seven types of drugs, and their occurrence frequency in these STs is 100%.

### Antibiotic resistance of pathogenic bacteria

Antibiotic resistance genes can be transferred from the environment to human pathogens through transformation, leading to resistance dissemination in microbial ecosystems, between different pathogen populations, and even across species (39). The tracking of spatiotemporal spread of specific antibiotic resistance genes can facilitate early detection and pathogen control (40,41).

**Figure 6.**
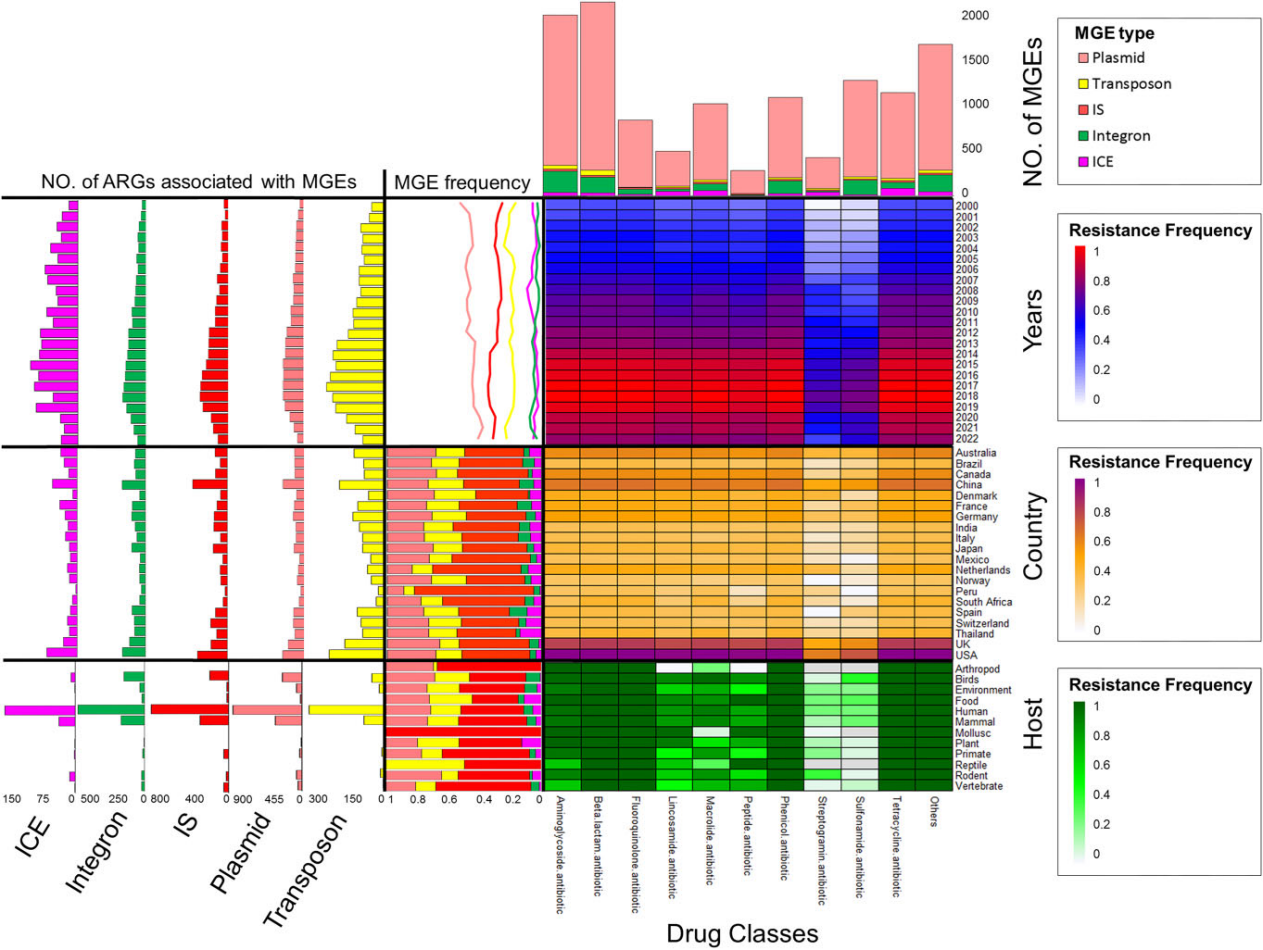
Resistance to different drug classes in 20 countries with the largest pathogen samples from 2000 to 2021. Pathogenic mobile genetic element frequencies and associated antibiotic resistance gene amounts in different years, countries and hosts are also shown.

As shown in Figure 6, the frequency of antibiotic resistance in most drug classes is increasing, indicating a serious problem with antibiotic resistance. Resistance to streptogramin and sulfonamide antibiotics remains lower compared to other drugs. Streptogramin is primarily used to treat tuberculosis, but the mutant gene *gidB* in *Mycobacterium tuberculosis* can reduce resistance by nearly 50%, potentially explaining the low frequency of resistance to streptogramin antibiotics (42). Additionally, sulfonamide antibiotics are more frequently used in veterinary practice than in human medicine, and the rapid development of sulfonamide resistance led to its later replacement by penicillin, resulting in decreased usage (43).

Moreover, the prevalence of antibiotic resistance genes is significantly higher in the USA and the UK, whereas data from South Africa and Peru show the lowest prevalence of such genes. Diverse medical standards and medication practices among different countries contribute to these variations. For instance, aminoglycoside apramycin has been used in the UK since 1978, leading to high resistance, especially in *Escherichia coli* (44).

### Mobile genetic elements of pathogenic bacteria

MGEs play a crucial role in bacterial horizontal transfer, enabling the acquisition of antibiotic resistance and virulence traits and facilitating adaptive evolution (30). Among the MGEs carrying ARGs annotated from genomic data, plasmids are the most prevalent, while integrative conjugative elements (ICE) carrying ARGs are the least common (Figure 6).

From 2000 to 2022, the frequency of MGE occurrence in pathogenic bacteria did not exhibit significant changes, but the number of ARGs associated with them increased (Figure 6). This finding, combined with the annual changes in ARG frequency, highlights the prominent role of MGEs in horizontal transfer of ARGs (30). Notably, plasmids, IS and transposons exhibited higher MGE frequencies than other types in the top 20 countries, with the USA, UK and China showing a higher number of ARGs associated with these MGEs. Plasmid-mediated horizontal transfer of ARGs is considered the most critical dissemination pathway of ARGs in humans, animals and the environment (45). Additionally, there were significant variations in the distribution of MGE frequency and associated ARG quantity among different host categories. Human-associated bacteria displayed a significantly higher number of ARGs associated with MGEs compared to other host categories, indicating that human-associated isolates experienced approximately 25-fold more horizontal gene transfer than non-human isolates (46).

## Conclusion and future perspective

gcPathogen harnesses big data on genomic sequencing, sequence types, epidemiology, antibiotic resistance, virulence factors and mobile genetic elements of human pathogens to support both scientific research and public health surveillance. The database will be regularly updated to include data on emerging pathogenic microorganisms from various sources. The growing volume of pathogen genome data from diverse hosts and geographical locations positions gcPathogen as a high-quality reference dataset, serving as a valuable data source for pan-genome and characteristic gene analysis, facilitating the design of novel genotyping markers for rapid and accurate target detection.

Currently, there is a gap in connecting pathogenetic data with traditional infectious disease surveillance systems. Integrating abundant phenotypic and genetic information of pathogens with various infection cases from clinical or disease control surveillance systems will aid in investigating the overall characteristics of pathogens, the transfer of drug-resistant genes and the spread of ‘super-resistant’ bacteria. Consequently, this integration will enhance surveillance measures and prevention activities. To address this, we plan to develop a user-friendly application programming interface (API) that will enable easy data connection with in-house and public infectious disease surveillance systems. This integration will translate data into real-time reports for decision-making by public health professionals and provide accurate early detection and warnings based on the integrated data.

## Supplementary Material

gkad875_Supplemental_File

## Data Availability

The data is freely accessible and downloadable from the platform through https://nmdc.cn/gcpathogen/download.
