# Supplementary material for: gcPathogen: a comprehensive genomic resource of human pathogens for public health
Source: Nucleic Acids Res. 2023 Oct 18;52(D1):D714–23. doi: 10.1093/nar/gkad875 (PMC10767814; doi:10.1093/nar/gkad875)
Supplement: gkad875_Supplemental_File [file gkad875_supplemental_file.docx]

**Supplementary Table**

**Table S1.** The cgMLST schemas constructed by gcPathogen and cited schemas from Enterobase, PubMLST, and PathogenWatch

| **Database names** | **Pathogen names** | **Tool taxon selection type** |
| --- | --- | --- |
| gcPathogen | Acinetobacter nosocomialis | Species |
|  | Acinetobacter pittii | Species |
|  | Aeromonas caviae | Species |
|  | Aeromonas dhakensis | Species |
|  | Aeromonas diversa | Species |
|  | Aeromonas hydrophila | Species |
|  | Aeromonas jandaei | Species |
|  | Aeromonas media | Species |
|  | Aeromonas schubertii | Species |
|  | Aeromonas veronii | Species |
|  | Anaplasmataceae | Genus |
|  | Bacillus anthracis | Species |
|  | Bacillus licheniformis | Species |
|  | Bacillus subtilis | Species |
|  | Bacillus thuringiensis | Species |
|  | Bacteroides fragilis | Species |
|  | Bacteroides ovatus | Species |
|  | Bartonella | Genus |
|  | Bordetella pertussis | Species |
|  | Burkholderia cenocepacia | Species |
|  | Burkholderia cepacia | Species |
|  | Burkholderia gladioli | Species |
|  | Burkholderia pseudomallei | Species |
|  | Campylobacter fetus | Species |
|  | Campylobacter lari | Species |
|  | Campylobacter upsaliensis | Species |
|  | Chlamydia trachomatis | Species |
|  | Citrobacter amalonaticus | Species |
|  | Citrobacter farmeri | Species |
|  | Citrobacter freundii | Species |
|  | Citrobacter koseri | Species |
|  | Citrobacter sedlakii | Species |
|  | Citrobacter youngae | Species |
|  | Citrobacter | Genus |
|  | Clostridium | Genus |
|  | Corynebacterium diphtheriae | Species |
|  | Corynebacterium striatum | Species |
|  | Cronobacter sakazakii | Species |
|  | Cutibacterium acnes | Species |
|  | Enterobacter asburiae | Species |
|  | Enterobacter cloacae | Species |
|  | Enterobacter hormaechei | Species |
|  | Enterobacter kobei | Species |
|  | Enterobacter roggenkampii | Species |
|  | Enterococcus faecalis | Species |
|  | Francisella tularensis | Species |
|  | Helicobacter_pylori | Species |
|  | Haemophilus influenzae | Species |
|  | Klebsiella aerogenes | Species |
|  | Klebsiella michiganensis | Species |
|  | Klebsiella oxytoca | Species |
|  | Lacticaseibacillus rhamnosus | Species |
|  | Lactiplantibacillus plantarum | Species |
|  | Lactococcus lactis | Species |
|  | Legionella pneumophila | Species |
|  | Leptospira interrogans | Species |
|  | Listeria innocua | Species |
|  | Listeria monocytogenes | Species |
|  | Mannheimia haemolytica | Species |
|  | Micrococcus luteus | Species |
|  | Moraxella_catarrhalis | Species |
|  | Morganella morganii | Species |
|  | Mycobacterium tuberculosis | Species |
|  | Pasteurella multocida | Species |
|  | Prevotella copri | Species |
|  | Proteus mirabilis | Species |
|  | Pseudomonas aeruginosa | Species |
|  | Serratia marcescens | Species |
|  | Staphylococcus argenteus | Species |
|  | Staphylococcus haemolyticus | Species |
|  | Stenotrophomonas maltophilia | Species |
|  | Treponema pallidum | Species |
| Enterobase | Salmonella | Genus |
|  | Escherichia/Shigella | Genus |
|  | Streptococcus | Species |
|  | Clostridioides | Species |
|  | Vibrio | Genus |
|  | Yersinia | Species |
| PubMLST | Acinetobacter baumannii | Species |
|  | Bacillus cereus | Species |
|  | Brucella | Genus |
|  | Burkholderia pseudomallei | Species |
|  | Campylobacter jejuni/coli | Species |
|  | Clostridium perfringens | Species |
|  | Neisseria | Species |
|  | Streptococcus pneumoniae | Species |
|  | Vibrio cholerae | Species |
| PathogenWatch | Acinetobacter baumannii | Species |
|  | Campylobacter jejuni/coli | Species |
|  | Enterococcus faecium | Species |
|  | Escherichia | Species |
|  | Klebsiella pneumoniae | Species |
|  | Klebsiella quasipneumoniae | Species |
|  | Klebsiella variicola | Species |
|  | Listeria | Species |
|  | Mycobacterium africanum | Species |
|  | Mycobacterium tuberculosis | Species |
|  | Neisseria gonorrhoeae | Species |
|  | Neisseria meningitides | Species |
|  | Salmonella | Genus |
|  | Shigella | Species |
|  | Staphylococcus aureus | Species |
|  | Vibrio cholerae | Species |

**Table S2.** The software and parameters used for MGE annotations.

| **Software (version)** | **Applicable MGE type** | **Parameters** | **Reference** |
| --- | --- | --- | --- |
| ISEScan (v1.7.2.3) | Insertion sequences (IS) | Remove incomplete IS sequences (--remove Short IS ), *evalue* ≤ 1e-5 | ([48](#_ENREF_48)) |
| DANMEL (v2.13.0+) | Insertion sequences (IS) | Identity > 90%, Scoverage > 80% | ([49](#_ENREF_49)) |
| MobileElementFinder (v1.1.1) | Insertion sequence (IS), transposons, integrative conjugative elements (ICE) | Coverage > 80%, *evalue* ≤ 1e-5, Identity > 90%, Scoverage > 80% | ([50](#_ENREF_50)) |
| ICEFinder (v1.0) | Integrative conjugative elements (ICE) | *evalue* ≤ 1e-5, Identity > 90%, Scoverage > 80% | ([51](#_ENREF_51)) |
| BacAnt (v3.4.0) | Transposons, integrons (IN) | Identity > 90%, Scoverage > 80% | ([52](#_ENREF_52)) |
| IntegronFinder2.0 (v2.0.2) | Plasmid | Remove Incomplete integrons (--local-max --func-annot) | ([53](#_ENREF_53)) |
| Platon (v1.6) | Plasmid | Sequence length < 2.5 Mb (--verbose) | ([54](#_ENREF_54)) |
| PlasmidFinder (v2.1.6.1) | Plasmid | Identity > 90%, Scoverage > 80% | ([55](#_ENREF_55)) |
